# Haplotype inference based on Hidden Markov Models in the QTL-MAS 2010 multi-generational dataset

**DOI:** 10.1186/1753-6561-5-S3-S10

**Published:** 2011-05-27

**Authors:** Carl Nettelblad

**Affiliations:** 1 Department of Information Technology, Uppsala University, Lägerhyddsvägen 2, Uppsala, Sweden

## Abstract

**Background:**

We have previously demonstrated an approach for efficient computation of genotype probabilities, and more generally probabilities of allele inheritance in inbred as well as outbred populations. That work also included an extension for haplotype inference, or phasing, using Hidden Markov Models. Computational phasing of multi-thousand marker datasets has not become common as of yet. In this communication, we further investigate the method presented earlier for such problems, in a multi-generational dataset simulated for QTL detection.

**Results:**

When analyzing the dataset simulated for the 14th QTLMAS workshop, the phasing produced showed zero deviations compared to original simulated phase in the founder generation. In total, 99.93% of all markers were correctly phased. 97.68% of the individuals were correct in all markers over all 5 simulated chromosomes. Results were produced over a weekend on a small computational cluster. The specific algorithmic adaptations needed for the Markov model training approach in order to reach convergence are described.

**Conclusions:**

Our method provides efficient, near-perfect haplotype inference allowing the determination of completely phased genomes in dense pedigrees. These developments are of special value for applications where marker alleles are not corresponding directly to QTL alleles, thus necessitating tracking of allele origin, and in complex multi-generational crosses. The cnF2freq codebase, which is in a current state of active development, is available under a BSD-style license.

## Background

Inference of haplotypes, or phasing, from genotype and pedigree data can be useful in several ways. For any kind of traditional linkage analysis, including QTL mapping, knowledge of haplotypes can help in producing a more correct analysis of linkage and thus higher statistical power and more well-defined positions. Knowledge of phase over the genome is also critical in an epigenetic context regarding sex-specific imprinting. Most of the research in reconstructing haplotypes from unphased data, like application of the EM algorithm [1], Clark’s algorithm [2], and certain Bayesian methods [3] were designed for cases where no pedigrees are available, and in some cases also with an assumption of Hardy-Weinberg equilibrium. Approaches based on MCMC (Markov Chain Monte Carlo) methods can integrate general pedigree information, but do so by sampling specific realisations, slowly improving the total haplotype solution. All these approaches tend to be prohibitively computationally expensive when the number of markers or individuals grows large. When pedigree data is known, rule-based heuristical approaches are also commonly suggested. These provide excellent performance, but will frequently fail to phase all positions, either leaving markers unphased, or selecting an incorrect resolution [4].

In this communication, we focus on reconstruction of haplotypes in experimental crosses of different designs. In such data, it can be expected that pedigree information is available and reliable. Furthermore, a large number of siblings (half or full) is generally available, making the direct tracking of recombination events an attractive way to infer haplotypes. A highly efficient method, with excellent convergence properties thanks to a specially adapted optimisation algorithm, is presented. This method has previously been briefly discussed with application to an earlier QTL-MAS workshop dataset [5] where it was shown to surpass the phasing results produced with other methods [6].

## Methods

A Hidden Markov Model (HMM) is defined by its states and transitions between states [7]. The hidden property is corresponding to the fact that states are not observed. Rather, emitted symbols in an alphabet are observed and those are related to the states according to some function.

### Review of Hidden Markov Models for intercross genotyping

Our haplotyping HMM approach is quite similar to the genotyping approach described for outbred lines in [5]. A first overview of the extension for haplotyping is also found there. A more formal description of the corresponding, slightly simpler, case for genotype probabilities in inbred lines can be found in [8], corresponding to the implementation found in R/qtl [9].

The state space in our model consists of a set of binary flags, determining the current phase in some individuals. In the case of finding genotype probabilities in an *F*_2_ cross, those flags represent the two *F*_1_ parent individuals, where the meiosis in each individual can transmit an allele originating from either the grandfather or the grandmother. Transitions between states represent recombination events. The Markov model is of the continuous-time variety, where time in this case corresponds to the mapping distance. Emission symbols correspond to the marker data. This constitutes the hidden quality of the model, as grandparental marker data might not map uniquely to the state.

### Application of HMMs for haplotyping

The genotype probability model in the intercross gains its speed from the fact that only a single “focus” individual, its parents and grandparents need to be analyzed at the same time. In the model, what is essentially tracked is the gametes generated by meiosis in the parents, expressed as grandparental origin in each locus. As two meioses are tracked, there are 2 ∗ 2 = 4 states.

The same general approach, where analysis takes place over a pedigree including a focus individual and the immediate ancestors to that focus individual, can be extended to include further generations of ancestors. Adding one more generation would imply 64 states (tracking 6 independent meioses), as even the phase in alleles that were not transmitted need to be calculated to accurately track possible state transitions. Adding another generation increases the number of states to 16, 384, which in practice is not tractable. As noted above, the genotyping model tracking phase in the parent, but that information is never recorded explicitly. In order to make the implicit tracking of phase in individual analyses explicit, we introduce a parameter, called skewness, per marker per individual. This parameter indicates the probability of the “true” ordering of the unordered marker value pair {*A*, *B*} to be *AB* or *BA*, respectively. Rather than simply using binary emission probabilities for supported or unsupported marker values, the emission probability will be dependent on the state realisation considered. A skewness parameter value of 0.5 is neutral to ordering, while a value of 0.0 exlusively permits *AB*, and a value of 1.0 will exclusively permit *BA*. A dataset is phased if the skewness parameters for all heterozygous marker value pairs take such extreme values.

For the haplotyping application, we are using the 64-state model with the addition of skewness parameters. The approach of centering each analysis on a focus individual is kept from the genotyping model. A full haplotyping iteration is thus only a matter of computing the model for each marker in each focus individual. Although no ancestral genotype information is used for the grandparents, the present of linkage will favour skewness assignments mapping linked alleles to the same strand. With a proper setup, the skewness parameters for all marker-individual pairs can be trained in an iterative manner based on analyses in the pedigrees where the individuals appear.

### Training methodology

We use a modified Baum-Welch approach [10] for training the skewness parameters. This approach is based on expectation-maximisation of the model probability for observed data under optimisation of some or all model parameters. The actual value distribution of a parameter under optimisation in one iterated realisation of the model is used as the prior distribution in the next one, until convergence is reached. The skewness parameter for the first heterozygote on each chromosome in each individual is fixed to 0.0, to avoid unnecessary symmetries. The transition parameters related to recombination can be kept fixed, based on a pre-determined marker map with (Haldane) mapping distances, or be subject to optimisation in tandem with the skewness parameter set.

The strand numbering is *not* directly connected to parental origin. Rather, the model is computed multiple times, allowing for the (grand)parental origin of each strand to be either the sire or dam for the respective parent. When the model has converged, most such alternatives can be truncated early due to extremely low likelihood, as a form of optimisation similar to a beam search. The total number of such shift combinations is 8 in the 64-state model, as shifts are only needed for the focus individual and its parents. The same skewness parameter is appearing in multiple separate analyses, as one individual can appear in multiple focus analyses: A parent will appear in the analysis pedigree of every offspring. An individual acting as the focus individual in one pedigree can also be a parent or a grandparent in another, in a multi-generational setting. Furthermore, some pedigrees can be completely uninformative for a specific parameter. The “optimal” skewness assignment based on such a pedigree will always be the a priori value of the parameter from the previous iteration, and it could be called skewness-agnostic. For these two reasons, a voting scheme is implemented. The skewness contribution from each analysis pedigree corresponds to the deviation from the a priori value used. Thus, uninformative locations are not hampering convergence if information is found in other pedigrees. Furthermore, the voting is scaled for contributions to ancestors more than one generation away from the focus individual. This is due to the fact that there is a single meiosis event truly tracked, the one resulting in a gamete forming the parent individual (*F*_1_). The offspring to that individual are only giving different aspects of information on the same event, and should be weighted accordingly. If this was not done, the haplotype estimates in an *F*_0_ individual would be biased towards the allele combination found in *F*_1_ individuals with a high number of *F*_2_ offspring.

The voting approach where lack of information is ignored was chosen to accelerate convergence. However, in early iterations information is lacking in many loci and those few fragments of information that are present can turn out to be incorrect. For this reason, the new value *p′* for the a skewness parameter with an a priori value of *p* is limited by clamping to satisfy the condition |*p* – *p′*| ≤ *p*(1 – *p*) min(1/(0.5 + (1 – *p*)), 1/(0.5 + *p*)). This clamping puts a limit to the relative change in preference of one phase assignment over the other, as expressed by the ratio *p*/(1 – *p*), to a factor of 3. For example, a skewness parameter value starting out at 0.5 will always be found in the range [0.25, 0.75] after one iteration. If the value is updated to 0.25, it will be found in the range [0.1, 0.5] in the following iteration. By limiting the rate of change for the probability ratio between these two complementary states, unstable behaviour is avoided.

In the *F*_0_ generation, the only definition of the alleles arises from the initialisation of the skewness value in one marker. In other words, the two strands can only be defined and separated relative to that anchor marker. Phasing a full chromosome, especially a long one, is problematic as the linkage between a distant marker and this anchor will be very weak or non-existent. The haplotyping problem based on meioses is inherently local, along the chromosome, and between closely related individuals. Skewness values might converge in different directions in different regions of the chromosome, assuming an extraneous recombination event. The traditional Baum-Welch approach, even with the modified voting scheme, would only resolve such a situation at a very slow pace, if at all. Therefore, an inversion step is added to the algorithm, where inverting all the skewness values downstream of the current marker is tested in each iteration.

If the total probability of the observed sequences, over all pedigrees, increases with such a change, it is accepted. Due to the model structure, such an inversion can be performed using the normal data structures of hidden Markov model algorithms, by essentially only modifying the state vector when combining forward and backward probabilities. In practice, care needs to be taken to avoid oscillatory behaviour, especially with respect to inversion events.

### Summarised algorithm

The following algorithm summarises the haplotyping approach described above for a single chromosome. As different chromosomes are completely unrelated from a linkage and phasing perspective, the whole approach can simply be repeated per chromosome. Isolating the phasing operation per chromosome can also simplify parallelisation of the computations and reduce the amount of memory needed for each step.

1. Initialise skewness values to 0.5 everywhere, except at one heterozygous marker in every individual

2. Loop until convergence (e.g. a minimum sum of skewness changes, or a fixed number of iterations based on chromosome length)

(a) Loop over all focus individuals

i. Loop over all markers

A. Compute the marginalised probability for strand realisations corresponding to observed marker values, taking all 8 parental shifts (defined above) into account, using suitable hidden Markov model algorithms.

B. Contribute the deviation between the current skewness value, and the resulting ratio, for each individual in the analysis pedigree, as a vote. Divide the votes to grandparents by the total number of (half-)siblings sharing that parent.

C. Compute and contribute probability changes arising from possible skewness inversions in each individual in the dataset in a similar manner.

(b) Loop over all individuals

i. Loop over all markers

A. Update skewness values according to recorded votes. Cap the maximum possible change in the ratio *p*/(1 – *p*) between iterations, in order to retain stability.

B. If the recorded votes for a favorable inversion at this position exceeds some threshold, perform an inversion of all downstream skewness values.

### Materials

The common dataset prepared for the 14th QTL-MAS workshop was used. This dataset contains 3,226 individuals, spread over 5 generations, with 20 founders. 10, 031 SNP markers were defined over 5 chromosomes all about 100 million base pairs in length. A uniform recombination rate of 1 cM/Mbp was applied for both sexes. Haplotypes for the founders were sampled from a simulated population of 5, 000, using the methods described in [11].

## Results

64 cores on Intel Core 2 Quad 2.66 GHz CPUs distributed over 8 nodes in a cluster were used for computations. The code is written in C++, parallelised using OpenMP and the MPI support in the Boost library [12]. Version 11.1 of the Intel C++ compiler was used with aggressive optimisation settings. One especially important consideration in this context is how the software and hardware stack as a whole handle so-called denormalised numbers, where we use truncation to zero to get adequate performance. Pseudo-asymptotic convergence was studied by completing 25 iterations in 3,116 minutes, whereas 10 iterations would suffice for almost identical results. The set of focus individuals were chosen to be the set of all non-founder individuals, thus including all genotype and ancestor information possible. The method was specified with 3 generations (64 states), so the individuals included in each local analysis pedigree included the focus individual, its parents and grandparents (if available).

**Figure 1:**
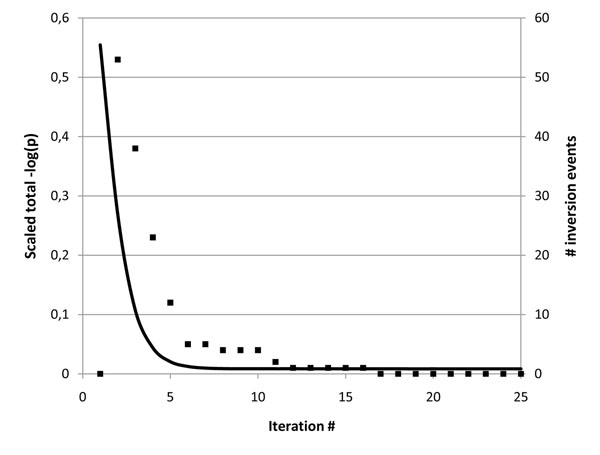
**Convergence**. Left axis (solid line) illustrating scaled sum of total logarithm probabilities over all analysis, up to iteration 25. In addition, block symbols (right vertical axis) show the number of downstream inversion events, most of which were occurring in the founder generation. After iteration 8, only minor adjustments were determined by the algorithm.

**Figure 2:**
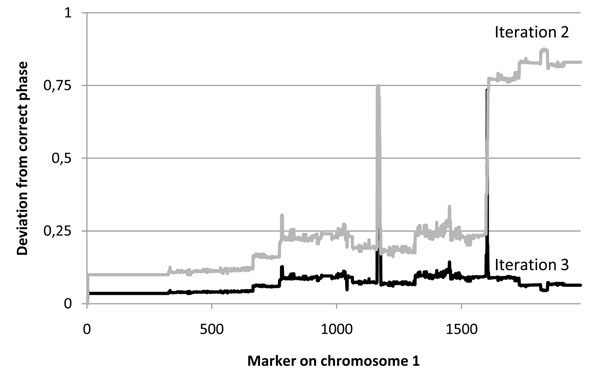
**Use of inversion**. During optimisation, a scan is performed in each analysis for inverting the skewness values determining phase downstream of each marker. Based on this scan, knots or bubbles of incorrect phase can be identified and resolved. The two lines are illustrating iteration 2 and 3, respectively, for chromosome 1 in the founder individual with ID 3. In iteration 2, a large range of markers has converged towards an inverted phase versus the correct one. This region is identified and inverted for iteration 3.

In an alternate test run with inversion turned off, convergence was not achieved within 25 iterations. Details on the rate of convergence, as well as the number of skewness inversion events per iteration, are illustrated in Figure 1. Inversions are in practice only needed during the very first few iterations, after which remaining uncertain skewness assignments slowly converge. The drastic improvement due to inversion is shown in Figure 2, where for the simplicity of presentation the true skewness value is illustrated as 0 over the full chromosome. The inversion scan locates subregions of the chromosome tending towards incompatible local optima and unifies them.

Results were compared for each generation separately. Generations were defined as contiguous blocks of numbered individuals in the dataset. In a few matings, a founder individual was mated to a generation 2 individual, but for our purposes (only relevant in the result presentation), the generation number of the resulting offspring is 3.

True haplotype information was provided by the organisers after the workshop. Comparisons demonstrate that correct phasing was achieved in 100% of heterozygous loci in the founder generation, where linkage in offspring was the only source of haplotype data. Total per-marker accuracy was over 99.9%, and over 99.5% in all generations. Other results are summarised per generation in Table 1.

**Table 1:** Accuracy per generation on the level of markers and individuals

Generation	Perfectly phased individuals	Correctly phased markers
1	100.0%	100.0%
2	93.78%	99.60%
3	97.69%	99.98%
4	98.67%	99.99%
5	98.33%	99.97%

All	97.68%	99.93%

Accuracy in produced phasing per generation. A perfectly phased individual has no heterozygotic marker with incorrect phase over the 5 chromosomes. Only a very limited amount of markers were incorrectly phased. The total proportion of individuals affected by errors was below 10% in all generations.

The distribution of phasing errors along chromosomes and between individuals is also relevant. Even in the most problematic generation, generation 2, over 93% of all individuals were phased correctly over all 10, 031 markers across 5 chromosomes, making the errors localised to a limited subset of the population.

## Discussion

In general, individuals with a rich pedigree have a higher probability of being phased correctly. This means that such individuals should preferably have parents and grandparents that themselves are correctly phased. In addition, a high number of *F*_1_ and *F*_2_ offspring are also of value. Each such individual represents a unique meiosis event. The linkage information available from multiple direct offspring, including further refinement through descendants in successive generations, gives much stronger information than relying on ancestry alone. Based on both of these conditions, generation 2 is at an disadvantage. Many individuals lack children altogether, and those that did procreate still have a limited number of grandchildren. They also have only parental genotype data, with no grandparental information. When long homozygous regions appear, there is no external reference (like linkage information from grandparents or grandchildren) to assist the phasing process.

The founder generation completely lacks ancestral genotype information, but this is compensated by the fact that every individual in the dataset is a descendant to the founders, and that founder reproductive success was reasonably evenly distributed. Therefore, every founder individual appeared in a high number of analysis pedigrees, giving the 100% correct result. The fact that each founder sire was mated to multiple dames also helped resolve possible ambiguities in both sexes, while in generation 2, most individuals with offspring only produced a single brood.

Efficient phasing of genotype data is a complex problem. Our presented approach provides excellent performance in a dataset that is representative of what can be encountered from high-quality genotyping in multi-generational crosses of heterogeneous stock. It should be noted that other methods achieving > 99% accuracy frequently do so by leaving some loci unphased. Our method will, on the other hand, converge in all positions, given proper initialisation. The most critical aspect is to choose a suitable locus for zero initialisation of skewness. If that locus can not be used to differentiate allele origin, the symmetry caused will result in the skewness values for *all* loci in that individual to remain unchanged. This situation typically arises if parents as well as grandparents are also heterozygous with the same alleles in the locus, with no known linkage, or if data is completely missing. In most cases, a sibling or cousin of the “unphasable” individual will contribute some information to the skewness values of parents and grandparents, eventually alleviating the symmetry, yielding convergence in all individuals. This was not a problem for the current dataset, but it has on occasion left individuals unphased in other datasets. Switching initalisation loci will generally remedy such cases.

The specific optimisation approach used is critical to attain the two conflicting goals of convergence within a reasonable number of iterations, as well as ensuring that the converged result is actually correct. The use of e.g. Viterbi training [13] would possibly accelerate the runtime, but the converged results would be highly inferior. In fact, such an approach would be more similar to existing heuristics-based solutions, as Viterbi training relies on the assumption that a single most likely realisation will dominate the results. In the early iterations, the exact opposite is true for the haplotyping problem, where a high number of different realisations of phasing are attributed very similar likelihood in the model.

## Conclusion

We have presented an efficient approach for determining phase from unphased marker data in a dense marker map, combined with pedigree data. In contrast to other approaches, we are able to take the full genotype information of the whole chromosome into account when determining optimal haplotypes. Thanks to the use of an efficient local Markov model, considering the direct ancestors to each individual, we are able to do this with high performance.

The quality and practical feasibility of our method for haplotype inference allow producing completely phased genomes in dense pedigrees, reducing the need for molecular methods for determining explicit haplotype data. The presence of phased data can be used to identify the origin of otherwise similar marker sequences, something that is needed to explore genetic architectures only indirectly connected to the marker map used (e.g. recent mutations in an SNP map general to the species studied).

In a simple dataset where genotyping errors are absent and no information is missing, the algorithm used could be greatly optimised by pruning the search tree, assigning probabilities of 0 rather than a small *ε* for improbable cases and assuming genotype data to be available for all positions. However, our main line for current work is rather to improve handling of missing and incorrect genotype data. For example, it is not uncommon that pedigree data is available even for individuals where genotyping failed or was never performed. Two specific cases under consideration are advanced line intercrosses and the analysis of heterogeneous stock.

The codebase did not undergo any specific changes to accommodate the workshop dataset, with the exception of modified routines for input and output in order to match relevant file formats. Code is available under a BSD-style license from our website, http://www.it.uu.se/research/project/ctrait. In parallel with further methods development, the introduction of a standardised R interface to the code is also underway.

## Competing interests

The author declares that he has no competing interests.
